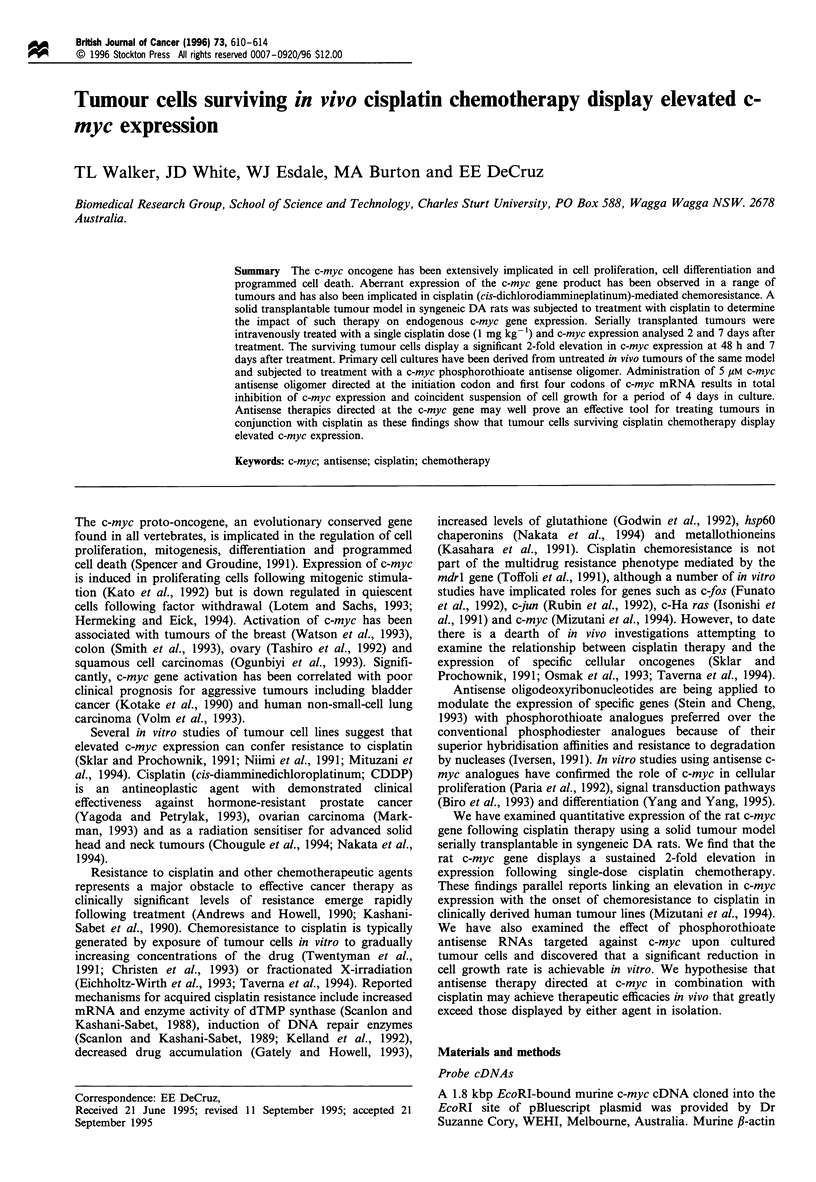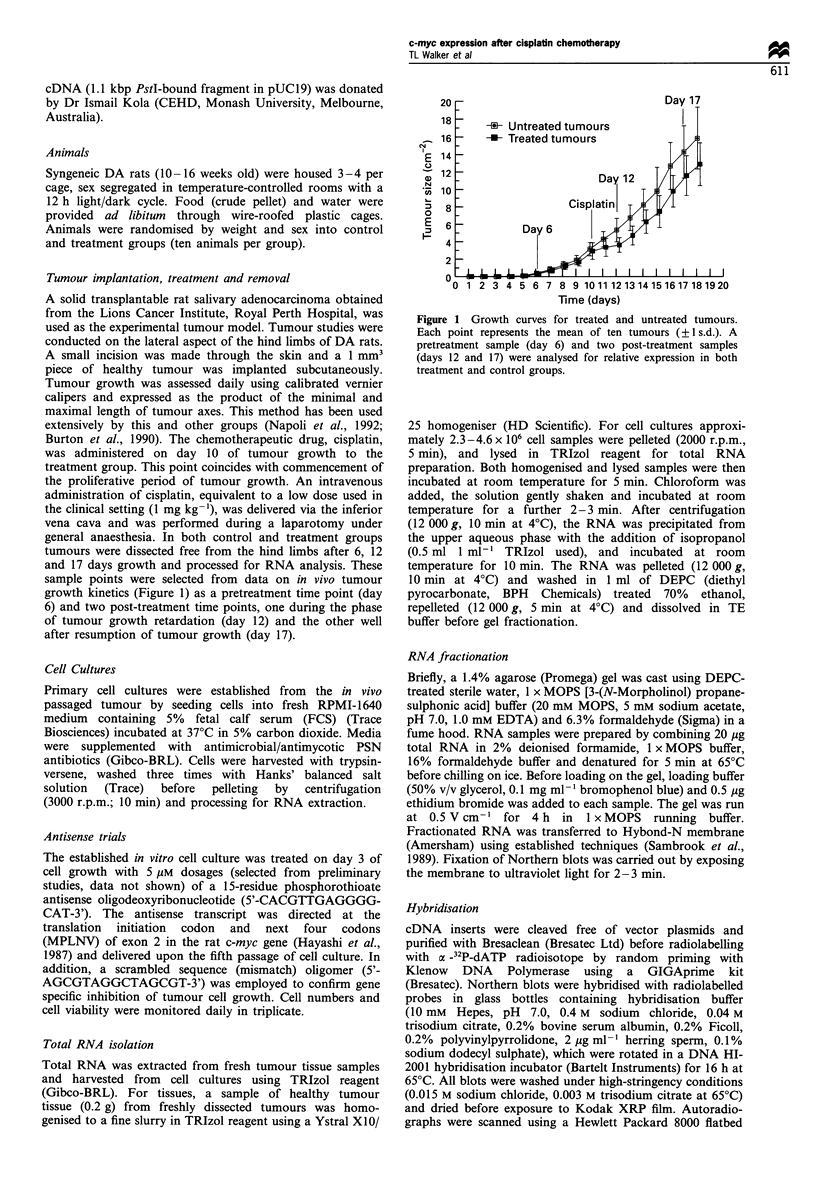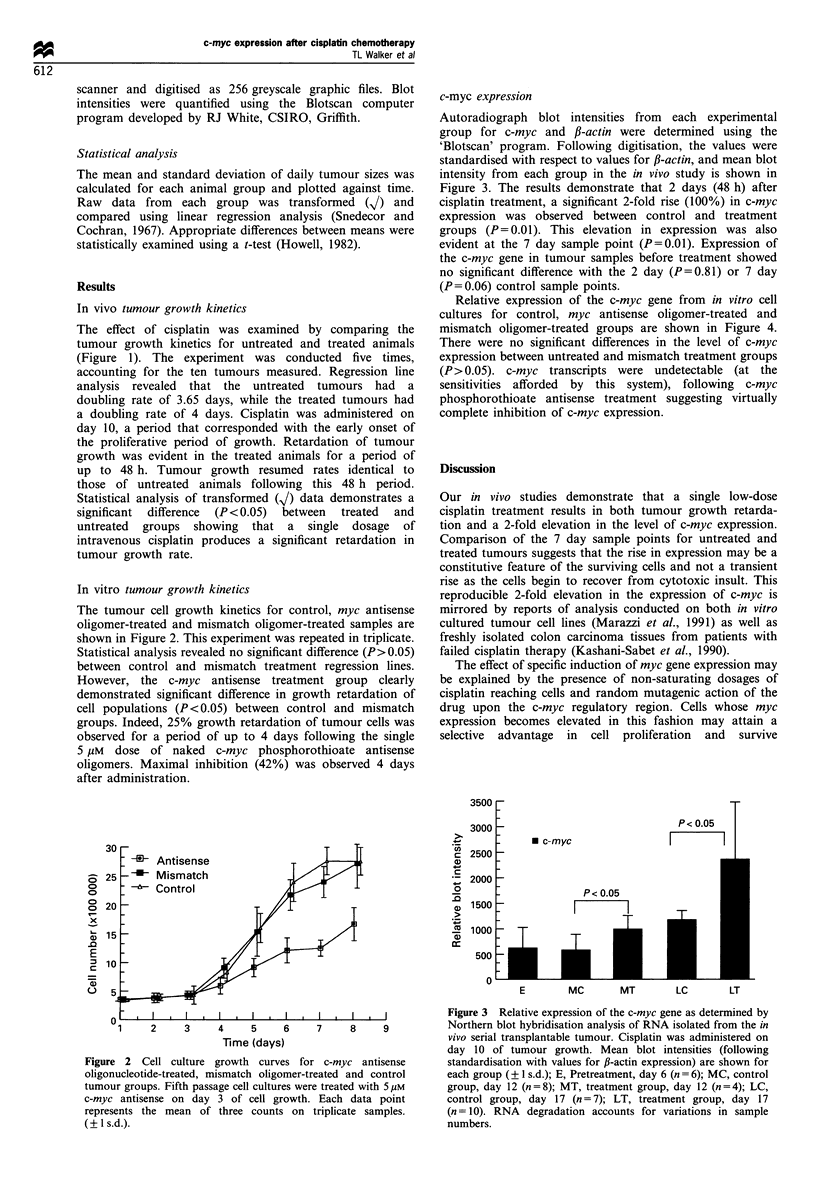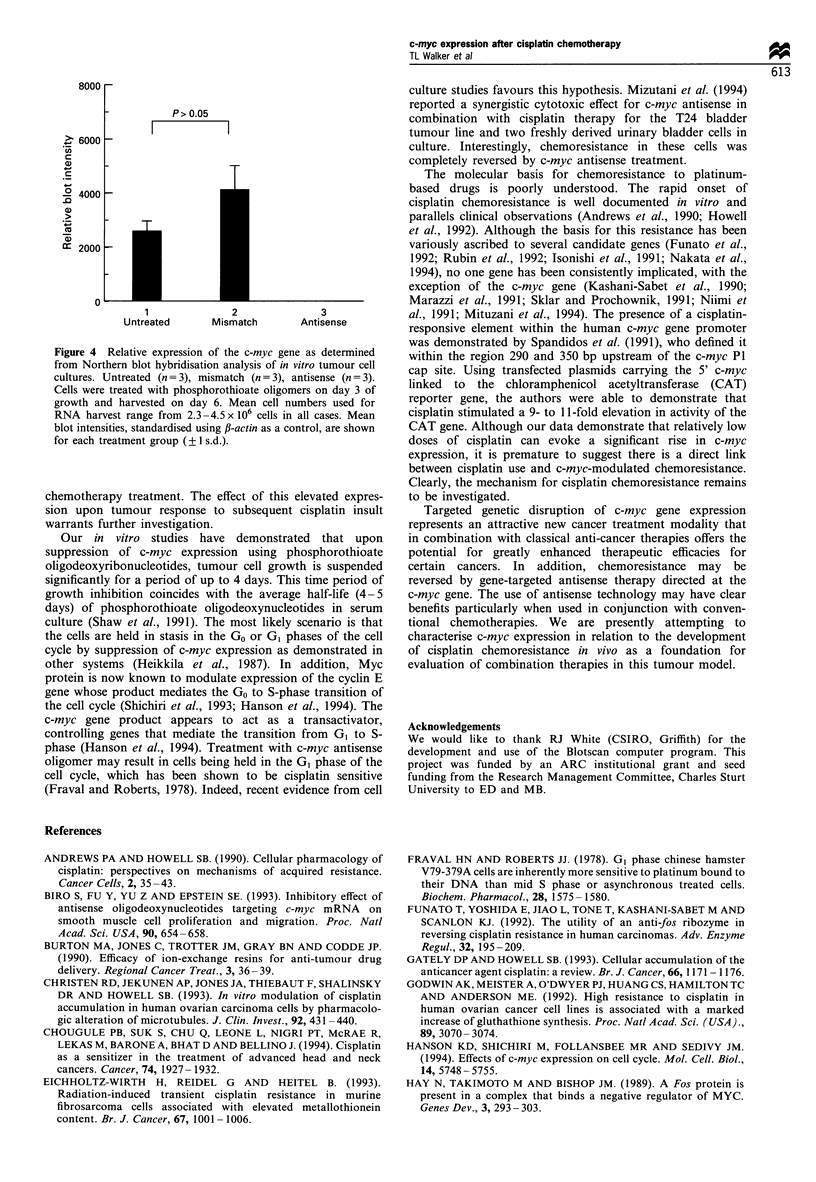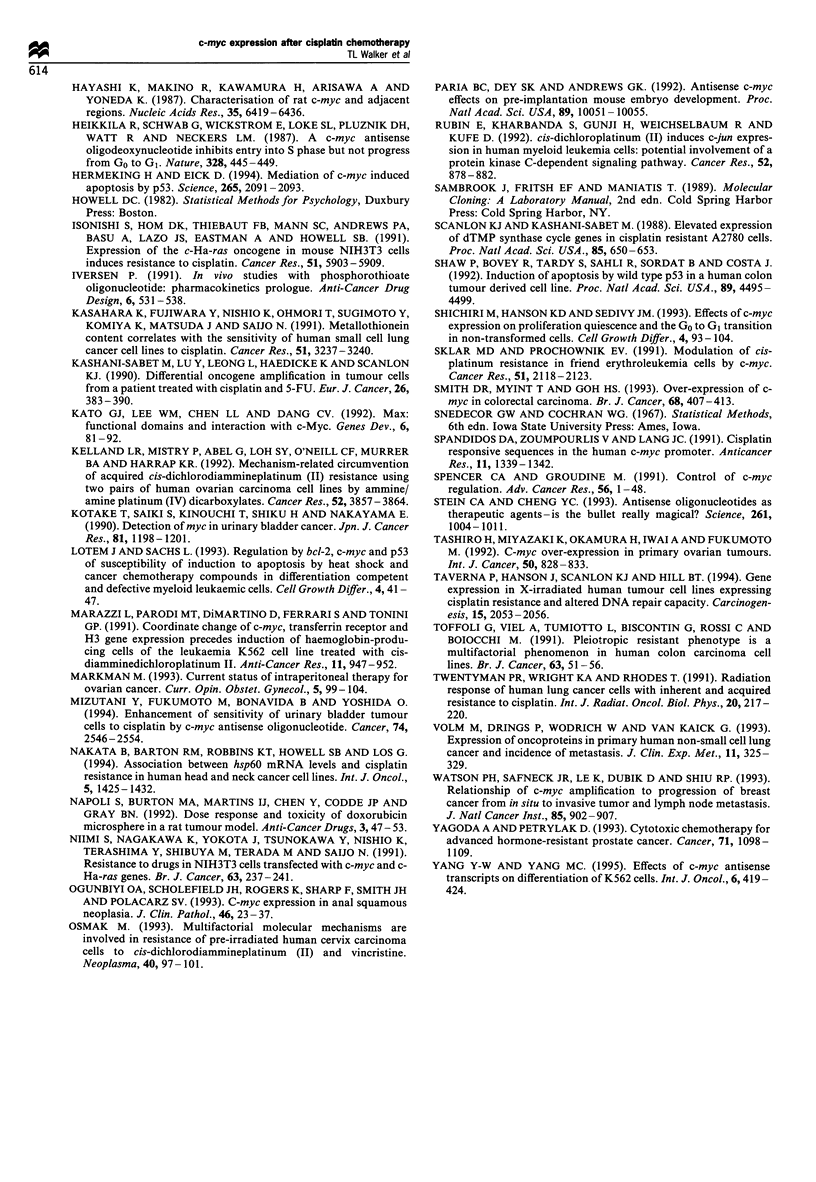# Tumour cells surviving in vivo cisplatin chemotherapy display elevated c-myc expression.

**DOI:** 10.1038/bjc.1996.105

**Published:** 1996-03

**Authors:** T. L. Walker, J. D. White, W. J. Esdale, M. A. Burton, E. E. DeCruz

**Affiliations:** Biomedical Research Group, School of Science and Technology, Charles Sturt University, Wagga Wagga NSW, Australia.

## Abstract

The c-myc oncogene has been extensively implicated in cell proliferation, cell differentiation and programmed cell death. Aberrant expression of the c-myc gene product has been observed in a range of tumours and has also been implicated in cisplatin (cis-dichlorodiammineplatinum)-mediated chemoresistance. A solid transplantable tumour model in syngeneic DA rats was subjected to treatment with cisplatin to determine the impact of such therapy on endogenous c-myc gene expression. Serially transplanted tumours were intravenously treated with a single cisplatin dose (1 mg/kg) and c-myc expression analysed 2 and 7 days after treatment. The surviving tumour cells display a significant 2-fold elevation in c-myc expression at 48 h and 7 days after treatment. Primary cell cultures have been derived from untreated in vivo tumours of the same model and subjected to treatment with a c-myc phosphorothioate antisense oligomer. Administration of 5 microM c-myc antisense oligomer directed at the initiation codon and first four codons of c-myc mRNA results in total inhibition of c-myc expression and coincident suspension of cell growth for a period of 4 days in culture. Antisense therapies directed at the c-myc gene may well prove an effective tool for treating tumours in conjunction with cisplatin as these findings show that tumour cells surviving cisplatin chemotherapy display elevated c-myc expression.


British Journal of Cancer (1996) 73, 610-614
© 1996 Stockton Press All rights reserved 0007-0920/96 $12.00

### Tumour cells surviving *in vivo* cisplatin chemotherapy display elevated c-*myc* expression

TL Walker, JD White, WJ Esdale, MA Burton and EE DeCruz

*Biomedical Research Group, School of Science and Technology, Charles Sturt University, PO Box 588, Wagga Wagga NSW. 2678 Australia.*

**Summary** The c-*myc* oncogene has been extensively implicated in cell proliferation, cell differentiation and programmed cell death. Aberrant expression of the c-*myc* gene product has been observed in a range of tumours and has also been implicated in cisplatin (*cis*-dichlorodiammineplatinum)-mediated chemoresistance. A solid transplantable tumour model in syngeneic DA rats was subjected to treatment with cisplatin to determine the impact of such therapy on endogenous c-*myc* gene expression. Serially transplanted tumours were intravenously treated with a single cisplatin dose (1 mg $kg^{-1}$) and c-*myc* expression analysed 2 and 7 days after treatment. The surviving tumour cells display a significant 2-fold elevation in c-*myc* expression at 48 h and 7 days after treatment. Primary cell cultures have been derived from untreated *in vivo* tumours of the same model and subjected to treatment with a c-*myc* phosphorothioate antisense oligomer. Administration of 5 $\mu$M c-*myc* antisense oligomer directed at the initiation codon and first four codons of c-*myc* mRNA results in total inhibition of c-*myc* expression and coincident suspension of cell growth for a period of 4 days in culture. Antisense therapies directed at the c-*myc* gene may well prove an effective tool for treating tumours in conjunction with cisplatin as these findings show that tumour cells surviving cisplatin chemotherapy display elevated c-*myc* expression.

**Keywords:** c-*myc*; antisense; cisplatin; chemotherapy

The c-*myc* proto-oncogene, an evolutionary conserved gene found in all vertebrates, is implicated in the regulation of cell proliferation, mitogenesis, differentiation and programmed cell death (Spencer and Groudine, 1991). Expression of c-*myc* is induced in proliferating cells following mitogenic stimulation (Kato *et al.*, 1992) but is down regulated in quiescent cells following factor withdrawal (Lotem and Sachs, 1993; Hermeking and Eick, 1994). Activation of c-*myc* has been associated with tumours of the breast (Watson *et al.*, 1993), colon (Smith *et al.*, 1993), ovary (Tashiro *et al.*, 1992) and squamous cell carcinomas (Ogunbiyi *et al.*, 1993). Significantly, c-*myc* gene activation has been correlated with poor clinical prognosis for aggressive tumours including bladder cancer (Kotake *et al.*, 1990) and human non-small-cell lung carcinoma (Volm *et al.*, 1993).

Several *in vitro* studies of tumour cell lines suggest that elevated c-*myc* expression can confer resistance to cisplatin (Sklar and Prochownik, 1991; Niimi *et al.*, 1991; Mituzani *et al.*, 1994). Cisplatin (*cis*-diamminedichloroplatinum; CDDP) is an antineoplastic agent with demonstrated clinical effectiveness against hormone-resistant prostate cancer (Yagoda and Petrylak, 1993), ovarian carcinoma (Markman, 1993) and as a radiation sensitiser for advanced solid head and neck tumours (Chougule *et al.*, 1994; Nakata *et al.*, 1994).

Resistance to cisplatin and other chemotherapeutic agents represents a major obstacle to effective cancer therapy as clinically significant levels of resistance emerge rapidly following treatment (Andrews and Howell, 1990; Kashani-Sabet *et al.*, 1990). Chemoresistance to cisplatin is typically generated by exposure of tumour cells *in vitro* to gradually increasing concentrations of the drug (Twentyman *et al.*, 1991; Christen *et al.*, 1993) or fractionated X-irradiation (Eichholtz-Wirth *et al.*, 1993; Taverna *et al.*, 1994). Reported mechanisms for acquired cisplatin resistance include increased mRNA and enzyme activity of dTMP synthase (Scanlon and Kashani-Sabet, 1988), induction of DNA repair enzymes (Scanlon and Kashani-Sabet, 1989; Kelland *et al.*, 1992), decreased drug accumulation (Gately and Howell, 1993), increased levels of glutathione (Godwin *et al.*, 1992), *hsp*60 chaperonins (Nakata *et al.*, 1994) and metallothioneins (Kasahara *et al.*, 1991). Cisplatin chemoresistance is not part of the multidrug resistance phenotype mediated by the *mdr*1 gene (Toffoli *et al.*, 1991), although a number of *in vitro* studies have implicated roles for genes such as c-*fos* (Funato *et al.*, 1992), c-*jun* (Rubin *et al.*, 1992), c-Ha *ras* (Isonishi *et al.*, 1991) and c-*myc* (Mizutani *et al.*, 1994). However, to date there is a dearth of *in vivo* investigations attempting to examine the relationship between cisplatin therapy and the expression of specific cellular oncogenes (Sklar and Prochownik, 1991; Osmak *et al.*, 1993; Taverna *et al.*, 1994).

Antisense oligodeoxyribonucleotides are being applied to modulate the expression of specific genes (Stein and Cheng, 1993) with phosphorothioate analogues preferred over the conventional phosphodiester analogues because of their superior hybridisation affinities and resistance to degradation by nucleases (Iversen, 1991). *In vitro* studies using antisense c-*myc* analogues have confirmed the role of c-*myc* in cellular proliferation (Paria *et al.*, 1992), signal transduction pathways (Biro *et al.*, 1993) and differentiation (Yang and Yang, 1995).

We have examined quantitative expression of the rat c-*myc* gene following cisplatin therapy using a solid tumour model serially transplantable in syngeneic DA rats. We find that the rat c-*myc* gene displays a sustained 2-fold elevation in expression following single-dose cisplatin chemotherapy. These findings parallel reports linking an elevation in c-*myc* expression with the onset of chemoresistance to cisplatin in clinically derived human tumour lines (Mizutani *et al.*, 1994). We have also examined the effect of phosphorothioate antisense RNAs targeted against c-*myc* upon cultured tumour cells and discovered that a significant reduction in cell growth rate is achievable *in vitro*. We hypothesise that antisense therapy directed at c-*myc* in combination with cisplatin may achieve therapeutic efficacies *in vivo* that greatly exceed those displayed by either agent in isolation.

### Materials and methods

### *Probe cDNAs*

A 1.8 kbp *Eco*RI-bound murine c-*myc* cDNA cloned into the *Eco*RI site of pBluescript plasmid was provided by Dr Suzanne Cory, WEHI, Melbourne, Australia. Murine $\beta$-actin

Correspondence: EE DeCruz,
Received 21 June 1995; revised 11 September 1995; accepted 21 September 1995

cDNA (1.1 kbp *Pst*I-bound fragment in pUC19) was donated by Dr Ismail Kola (CEHD, Monash University, Melbourne, Australia).

*Animals*

Syngeneic DA rats (10–16 weeks old) were housed 3–4 per cage, sex segregated in temperature-controlled rooms with a 12 h light/dark cycle. Food (crude pellet) and water were provided *ad libitum* through wire-roofed plastic cages. Animals were randomised by weight and sex into control and treatment groups (ten animals per group).

*Tumour implantation, treatment and removal*

A solid transplantable rat salivary adenocarcinoma obtained from the Lions Cancer Institute, Royal Perth Hospital, was used as the experimental tumour model. Tumour studies were conducted on the lateral aspect of the hind limbs of DA rats. A small incision was made through the skin and a 1 $mm^3$ piece of healthy tumour was implanted subcutaneously. Tumour growth was assessed daily using calibrated vernier calipers and expressed as the product of the minimal and maximal length of tumour axes. This method has been used extensively by this and other groups (Napoli *et al.*, 1992; Burton *et al.*, 1990). The chemotherapeutic drug, cisplatin, was administered on day 10 of tumour growth to the treatment group. This point coincides with commencement of the proliferative period of tumour growth. An intravenous administration of cisplatin, equivalent to a low dose used in the clinical setting (1 mg $kg^{-1}$), was delivered via the inferior vena cava and was performed during a laparotomy under general anaesthesia. In both control and treatment groups tumours were dissected free from the hind limbs after 6, 12 and 17 days growth and processed for RNA analysis. These sample points were selected from data on *in vivo* tumour growth kinetics (Figure 1) as a pretreatment time point (day 6) and two post-treatment time points, one during the phase of tumour growth retardation (day 12) and the other well after resumption of tumour growth (day 17).

*Cell Cultures*

Primary cell cultures were established from the *in vivo* passaged tumour by seeding cells into fresh RPMI-1640 medium containing 5% fetal calf serum (FCS) (Trace Biosciences) incubated at 37°C in 5% carbon dioxide. Media were supplemented with antimicrobial/antimycotic PSN antibiotics (Gibco-BRL). Cells were harvested with trypsin-versene, washed three times with Hanks' balanced salt solution (Trace) before pelleting by centrifugation (3000 r.p.m.; 10 min) and processing for RNA extraction.

*Antisense trials*

The established *in vitro* cell culture was treated on day 3 of cell growth with 5 $\mu$M dosages (selected from preliminary studies, data not shown) of a 15-residue phosphorothioate antisense oligodeoxyribonucleotide (5'-CACGTTGAGGGG-CAT-3'). The antisense transcript was directed at the translation initiation codon and next four codons (MPLNV) of exon 2 in the rat c-*myc* gene (Hayashi *et al.*, 1987) and delivered upon the fifth passage of cell culture. In addition, a scrambled sequence (mismatch) oligomer (5'-AGCGTAGGCTAGCGT-3') was employed to confirm gene specific inhibition of tumour cell growth. Cell numbers and cell viability were monitored daily in triplicate.

*Total RNA isolation*

Total RNA was extracted from fresh tumour tissue samples and harvested from cell cultures using TRIzol reagent (Gibco-BRL). For tissues, a sample of healthy tumour tissue (0.2 g) from freshly dissected tumours was homogenised to a fine slurry in TRIzol reagent using a Ystral X10/

**Figure 1** Growth curves for treated and untreated tumours. Each point represents the mean of ten tumours (±1 s.d.). A pretreatment sample (day 6) and two post-treatment samples (days 12 and 17) were analysed for relative expression in both treatment and control groups.

25 homogeniser (HD Scientific). For cell cultures approximately 2.3–4.6 × $10^6$ cell samples were pelleted (2000 r.p.m., 5 min), and lysed in TRIzol reagent for total RNA preparation. Both homogenised and lysed samples were then incubated at room temperature for 5 min. Chloroform was added, the solution gently shaken and incubated at room temperature for a further 2–3 min. After centrifugation (12 000 ***g***, 10 min at 4°C), the RNA was precipitated from the upper aqueous phase with the addition of isopropanol (0.5 ml 1 $ml^{-1}$ TRIzol used), and incubated at room temperature for 10 min. The RNA was pelleted (12 000 ***g***, 10 min at 4°C) and washed in 1 ml of DEPC (diethyl pyrocarbonate, BPH Chemicals) treated 70% ethanol, repelleted (12 000 ***g***, 5 min at 4°C) and dissolved in TE buffer before gel fractionation.

*RNA fractionation*

Briefly, a 1.4% agarose (Promega) gel was cast using DEPC-treated sterile water, 1 × MOPS [3-(*N*-Morpholinol) propane-sulphonic acid] buffer (20 mM MOPS, 5 mM sodium acetate, pH 7.0, 1.0 mM EDTA) and 6.3% formaldehyde (Sigma) in a fume hood. RNA samples were prepared by combining 20 $\mu$g total RNA in 2% deionised formamide, 1 × MOPS buffer, 16% formaldehyde buffer and denatured for 5 min at 65°C before chilling on ice. Before loading on the gel, loading buffer (50% v/v glycerol, 0.1 mg $ml^{-1}$ bromophenol blue) and 0.5 $\mu$g ethidium bromide was added to each sample. The gel was run at 0.5 V $cm^{-1}$ for 4 h in 1 × MOPS running buffer. Fractionated RNA was transferred to Hybond-N membrane (Amersham) using established techniques (Sambrook *et al.*, 1989). Fixation of Northern blots was carried out by exposing the membrane to ultraviolet light for 2–3 min.

*Hybridisation*

cDNA inserts were cleaved free of vector plasmids and purified with Bresaclean (Bresatec Ltd) before radiolabelling with $\alpha$-$^{32}$P-dATP radioisotope by random priming with Klenow DNA Polymerase using a GIGAprime kit (Bresatec). Northern blots were hybridised with radiolabelled probes in glass bottles containing hybridisation buffer (10 mM Hepes, pH 7.0, 0.4 M sodium chloride, 0.04 M trisodium citrate, 0.2% bovine serum albumin, 0.2% Ficoll, 0.2% polyvinylpyrrolidone, 2 $\mu$g $ml^{-1}$ herring sperm, 0.1% sodium dodecyl sulphate), which were rotated in a DNA HI-2001 hybridisation incubator (Bartelt Instruments) for 16 h at 65°C. All blots were washed under high-stringency conditions (0.015 M sodium chloride, 0.003 M trisodium citrate at 65°C) and dried before exposure to Kodak XRP film. Autoradiographs were scanned using a Hewlett Packard 8000 flatbed scanner and digitised as 256 greyscale graphic files. Blot intensities were quantified using the Blotscan computer program developed by RJ White, CSIRO, Griffith.

*Statistical analysis*

The mean and standard deviation of daily tumour sizes was calculated for each animal group and plotted against time. Raw data from each group was transformed ($\surd$) and compared using linear regression analysis (Snedecor and Cochran, 1967). Appropriate differences between means were statistically examined using a *t*-test (Howell, 1982).

### Results

In vivo *tumour growth kinetics*

The effect of cisplatin was examined by comparing the tumour growth kinetics for untreated and treated animals (Figure 1). The experiment was conducted five times, accounting for the ten tumours measured. Regression line analysis revealed that the untreated tumours had a doubling rate of 3.65 days, while the treated tumours had a doubling rate of 4 days. Cisplatin was administered on day 10, a period that corresponded with the early onset of the proliferative period of growth. Retardation of tumour growth was evident in the treated animals for a period of up to 48 h. Tumour growth resumed rates identical to those of untreated animals following this 48 h period. Statistical analysis of transformed ($\surd$) data demonstrates a significant difference ($P<0.05$) between treated and untreated groups showing that a single dosage of intravenous cisplatin produces a significant retardation in tumour growth rate.

In vitro *tumour growth kinetics*

The tumour cell growth kinetics for control, *myc* antisense oligomer-treated and mismatch oligomer-treated samples are shown in Figure 2. This experiment was repeated in triplicate. Statistical analysis revealed no significant difference ($P>0.05$) between control and mismatch treatment regression lines. However, the c-*myc* antisense treatment group clearly demonstrated significant difference in growth retardation of cell populations ($P<0.05$) between control and mismatch groups. Indeed, 25% growth retardation of tumour cells was observed for a period of up to 4 days following the single 5 $\mu$M dose of naked c-*myc* phosphorothioate antisense oligomers. Maximal inhibition (42%) was observed 4 days after administration.

c-myc *expression*

Autoradiograph blot intensities from each experimental group for c-*myc* and *β-actin* were determined using the 'Blotscan' program. Following digitisation, the values were standardised with respect to values for *β-actin*, and mean blot intensity from each group in the *in vivo* study is shown in Figure 3. The results demonstrate that 2 days (48 h) after cisplatin treatment, a significant 2-fold rise (100%) in c-*myc* expression was observed between control and treatment groups ($P=0.01$). This elevation in expression was also evident at the 7 day sample point ($P=0.01$). Expression of the c-*myc* gene in tumour samples before treatment showed no significant difference with the 2 day ($P=0.81$) or 7 day ($P=0.06$) control sample points.

Relative expression of the c-*myc* gene from *in vitro* cell cultures for control, *myc* antisense oligomer-treated and mismatch oligomer-treated groups are shown in Figure 4. There were no significant differences in the level of c-*myc* expression between untreated and mismatch treatment groups ($P>0.05$). c-*myc* transcripts were undetectable (at the sensitivities afforded by this system), following c-*myc* phosphorothioate antisense treatment suggesting virtually complete inhibition of c-*myc* expression.

### Discussion

Our *in vivo* studies demonstrate that a single low-dose cisplatin treatment results in both tumour growth retardation and a 2-fold elevation in the level of c-*myc* expression. Comparison of the 7 day sample points for untreated and treated tumours suggests that the rise in expression may be a constitutive feature of the surviving cells and not a transient rise as the cells begin to recover from cytotoxic insult. This reproducible 2-fold elevation in the expression of c-*myc* is mirrored by reports of analysis conducted on both *in vitro* cultured tumour cell lines (Marazzi *et al.*, 1991) as well as freshly isolated colon carcinoma tissues from patients with failed cisplatin therapy (Kashani-Sabet *et al.*, 1990).

The effect of specific induction of *myc* gene expression may be explained by the presence of non-saturating dosages of cisplatin reaching cells and random mutagenic action of the drug upon the c-*myc* regulatory region. Cells whose *myc* expression becomes elevated in this fashion may attain a selective advantage in cell proliferation and survive

**Figure 2** Cell culture growth curves for c-*myc* antisense oligonucleotide-treated, mismatch oligomer-treated and control tumour groups. Fifth passage cell cultures were treated with 5 $\mu$M c-*myc* antisense on day 3 of cell growth. Each data point represents the mean of three counts on triplicate samples. ($\pm$1 s.d.).

**Figure 3** Relative expression of the c-*myc* gene as determined by Northern blot hybridisation analysis of RNA isolated from the *in vivo* serial transplantable tumour. Cisplatin was administered on day 10 of tumour growth. Mean blot intensities (following standardisation with values for *β*-actin expression) are shown for each group ($\pm$1 s.d.); E, Pretreatment, day 6 ($n=6$); MC, control group, day 12 ($n=8$); MT, treatment group, day 12 ($n=4$); LC, control group, day 17 ($n=7$); LT, treatment group, day 17 ($n=10$). RNA degradation accounts for variations in sample numbers.

**Figure 4** Relative expression of the c-*myc* gene as determined from Northern blot hybridisation analysis of *in vitro* tumour cell cultures. Untreated ($n=3$), mismatch ($n=3$), antisense ($n=3$). Cells were treated with phosphorothioate oligomers on day 3 of growth and harvested on day 6. Mean cell numbers used for RNA harvest range from $2.3-4.5 \times 10^6$ cells in all cases. Mean blot intensities, standardised using *β-actin* as a control, are shown for each treatment group ($\pm 1$ s.d.).

chemotherapy treatment. The effect of this elevated expression upon tumour response to subsequent cisplatin insult warrants further investigation.

Our *in vitro* studies have demonstrated that upon suppression of c-*myc* expression using phosphorothioate oligodeoxyribonucleotides, tumour cell growth is suspended significantly for a period of up to 4 days. This time period of growth inhibition coincides with the average half-life (4–5 days) of phosphorothioate oligodeoxynucleotides in serum culture (Shaw *et al.*, 1991). The most likely scenario is that the cells are held in stasis in the $G_0$ or $G_1$ phases of the cell cycle by suppression of c-*myc* expression as demonstrated in other systems (Heikkila *et al.*, 1987). In addition, Myc protein is now known to modulate expression of the cyclin E gene whose product mediates the $G_0$ to S-phase transition of the cell cycle (Shichiri *et al.*, 1993; Hanson *et al.*, 1994). The c-*myc* gene product appears to act as a transactivator, controlling genes that mediate the transition from $G_1$ to S-phase (Hanson *et al.*, 1994). Treatment with c-*myc* antisense oligomer may result in cells being held in the $G_1$ phase of the cell cycle, which has been shown to be cisplatin sensitive (Fraval and Roberts, 1978). Indeed, recent evidence from cell culture studies favours this hypothesis. Mizutani *et al.* (1994) reported a synergistic cytotoxic effect for c-*myc* antisense in combination with cisplatin therapy for the T24 bladder tumour line and two freshly derived urinary bladder cells in culture. Interestingly, chemoresistance in these cells was completely reversed by c-*myc* antisense treatment.

The molecular basis for chemoresistance to platinum-based drugs is poorly understood. The rapid onset of cisplatin chemoresistance is well documented *in vitro* and parallels clinical observations (Andrews *et al.*, 1990; Howell *et al.*, 1992). Although the basis for this resistance has been variously ascribed to several candidate genes (Funato *et al.*, 1992; Rubin *et al.*, 1992; Isonishi *et al.*, 1991; Nakata *et al.*, 1994), no one gene has been consistently implicated, with the exception of the c-*myc* gene (Kashani-Sabet *et al.*, 1990; Marazzi *et al.*, 1991; Sklar and Prochownik, 1991; Niimi *et al.*, 1991; Mituzani *et al.*, 1994). The presence of a cisplatin-responsive element within the human c-*myc* gene promoter was demonstrated by Spandidos *et al.* (1991), who defined it within the region 290 and 350 bp upstream of the c-*myc* P1 cap site. Using transfected plasmids carrying the 5' c-*myc* linked to the chloramphenicol acetyltransferase (CAT) reporter gene, the authors were able to demonstrate that cisplatin stimulated a 9- to 11-fold elevation in activity of the CAT gene. Although our data demonstrate that relatively low doses of cisplatin can evoke a significant rise in c-*myc* expression, it is premature to suggest there is a direct link between cisplatin use and c-*myc*-modulated chemoresistance. Clearly, the mechanism for cisplatin chemoresistance remains to be investigated.

Targeted genetic disruption of c-*myc* gene expression represents an attractive new cancer treatment modality that in combination with classical anti-cancer therapies offers the potential for greatly enhanced therapeutic efficacies for certain cancers. In addition, chemoresistance may be reversed by gene-targeted antisense therapy directed at the c-*myc* gene. The use of antisense technology may have clear benefits particularly when used in conjunction with conventional chemotherapies. We are presently attempting to characterise c-*myc* expression in relation to the development of cisplatin chemoresistance *in vivo* as a foundation for evaluation of combination therapies in this tumour model.

**Acknowledgements**

We would like to thank RJ White (CSIRO, Griffith) for the development and use of the Blotscan computer program. This project was funded by an ARC institutional grant and seed funding from the Research Management Committee, Charles Sturt University to ED and MB.

### References

ANDREWS PA AND HOWELL SB. (1990). Cellular pharmacology of cisplatin: perspectives on mechanisms of acquired resistance. *Cancer Cells*, **2**, 35–43.

BIRO S, FU Y, YU Z AND EPSTEIN SE. (1993). Inhibitory effect of antisense oligodeoxynucleotides targeting *c-myc* mRNA on smooth muscle cell proliferation and migration. *Proc. Natl Acad. Sci. USA*, **90**, 654–658.

BURTON MA, JONES C, TROTTER JM, GRAY BN AND CODDE JP. (1990). Efficacy of ion-exchange resins for anti-tumour drug delivery. *Regional Cancer Treat.*, **3**, 36–39.

CHRISTEN RD, JEKUNEN AP, JONES JA, THIEBAUT F, SHALINSKY DR AND HOWELL SB. (1993). *In vitro* modulation of cisplatin accumulation in human ovarian carcinoma cells by pharmacologic alteration of microtubules. *J. Clin. Invest.*, **92**, 431–440.

CHOUGULE PB, SUK S, CHU Q, LEONE L, NIGRI PT, McRAE R, LEKAS M, BARONE A, BHAT D AND BELLINO J. (1994). Cisplatin as a sensitizer in the treatment of advanced head and neck cancers. *Cancer*, **74**, 1927–1932.

EICHHOLTZ-WIRTH H, REIDEL G AND HEITEL B. (1993). Radiation-induced transient cisplatin resistance in murine fibrosarcoma cells associated with elevated metallothionein content. *Br. J. Cancer*, **67**, 1001–1006.

FRAVAL HN AND ROBERTS JJ. (1978). $G_1$ phase chinese hamster V79-379A cells are inherently more sensitive to platinum bound to their DNA than mid S phase or asynchronous treated cells. *Biochem. Pharmacol.*, **28**, 1575–1580.

FUNATO T, YOSHIDA E, JIAO L, TONE T, KASHANI-SABET M AND SCANLON KJ. (1992). The utility of an anti-*fos* ribozyme in reversing cisplatin resistance in human carcinomas. *Adv. Enzyme Regul.*, **32**, 195–209.

GATELY DP AND HOWELL SB. (1993). Cellular accumulation of the anticancer agent cisplatin: a review. *Br. J. Cancer*, **66**, 1171–1176.

GODWIN AK, MEISTER A, O'DWYER PJ, HUANG CS, HAMILTON TC AND ANDERSON ME. (1992). High resistance to cisplatin in human ovarian cancer cell lines is associated with a marked increase of gluthathione synthesis. *Proc. Natl Acad. Sci. (USA).*, **89**, 3070–3074.

HANSON KD, SHICHIRI M, FOLLANSBEE MR AND SEDIVY JM. (1994). Effects of c-*myc* expression on cell cycle. *Mol. Cell. Biol.*, **14**, 5748–5755.

HAY N, TAKIMOTO M AND BISHOP JM. (1989). A *Fos* protein is present in a complex that binds a negative regulator of MYC. *Genes Dev.*, **3**, 293–303.

HAYASHI K, MAKINO R, KAWAMURA H, ARISAWA A AND YONEDA K. (1987). Characterisation of rat c-*myc* and adjacent regions. *Nucleic Acids Res.*, **35**, 6419–6436.

HEIKKILA R, SCHWAB G, WICKSTROM E, LOKE SL, PLUZNIK DH, WATT R AND NECKERS LM. (1987). A c-*myc* antisense oligodeoxynucleotide inhibits entry into S phase but not progress from $G_0$ to $G_1$. *Nature*, **328**, 445–449.

HERMEKING H AND EICK D. (1994). Mediation of c-*myc* induced apoptosis by p53. *Science*, **265**, 2091–2093.

HOWELL DC. (1982). *Statistical Methods for Psychology*, Duxbury Press: Boston.

ISONISHI S, HOM DK, THIEBAUT FB, MANN SC, ANDREWS PA, BASU A, LAZO JS, EASTMAN A AND HOWELL SB. (1991). Expression of the *c*-Ha-*ras* oncogene in mouse NIH3T3 cells induces resistance to cisplatin. *Cancer Res.*, **51**, 5903–5909.

IVERSEN P. (1991). *In vivo* studies with phosphorothioate oligonucleotide: pharmacokinetics prologue. *Anti-Cancer Drug Design*, **6**, 531–538.

KASAHARA K, FUJIWARA Y, NISHIO K, OHMORI T, SUGIMOTO Y, KOMIYA K, MATSUDA J AND SAIJO N. (1991). Metallothionein content correlates with the sensitivity of human small cell lung cancer cell lines to cisplatin. *Cancer Res.*, **51**, 3237–3240.

KASHANI-SABET M, LU Y, LEONG L, HAEDICKE K AND SCANLON KJ. (1990). Differential oncogene amplification in tumour cells from a patient treated with cisplatin and 5-FU. *Eur. J. Cancer*, **26**, 383–390.

KATO GJ, LEE WM, CHEN LL AND DANG CV. (1992). Max: functional domains and interaction with c-Myc. *Genes Dev.*, **6**, 81–92.

KELLAND LR, MISTRY P, ABEL G, LOH SY, O'NEILL CF, MURRER BA AND HARRAP KR. (1992). Mechanism-related circumvention of acquired *cis*-dichlorodiammineplatinum (II) resistance using two pairs of human ovarian carcinoma cell lines by ammine/amine platinum (IV) dicarboxylates. *Cancer Res.*, **52**, 3857–3864.

KOTAKE T, SAIKI S, KINOUCHI T, SHIKU H AND NAKAYAMA E. (1990). Detection of *myc* in urinary bladder cancer. *Jpn. J. Cancer Res.*, **81**, 1198–1201.

LOTEM J AND SACHS L. (1993). Regulation by *bcl*-2, c-*myc* and p53 of susceptibility of induction to apoptosis by heat shock and cancer chemotherapy compounds in differentiation competent and defective myeloid leukaemic cells. *Cell Growth Differ.*, **4**, 41–47.

MARAZZI L, PARODI MT, DiMARTINO D, FERRARI S AND TONINI GP. (1991). Coordinate change of c-*myc*, transferrin receptor and H3 gene expression precedes induction of haemoglobin-producing cells of the leukaemia K562 cell line treated with cis-diamminedichloroplatinum II. *Anti-Cancer Res.*, **11**, 947–952.

MARKMAN M. (1993). Current status of intraperitoneal therapy for ovarian cancer. *Curr. Opin. Obstet. Gynecol.*, **5**, 99–104.

MIZUTANI Y, FUKUMOTO M, BONAVIDA B AND YOSHIDA O. (1994). Enhancement of sensitivity of urinary bladder tumour cells to cisplatin by c-*myc* antisense oligonucleotide. *Cancer*, **74**, 2546–2554.

NAKATA B, BARTON RM, ROBBINS KT, HOWELL SB AND LOS G. (1994). Association between *hsp*60 mRNA levels and cisplatin resistance in human head and neck cancer cell lines. *Int. J. Oncol.*, **5**, 1425–1432.

NAPOLI S, BURTON MA, MARTINS IJ, CHEN Y, CODDE JP AND GRAY BN. (1992). Dose response and toxicity of doxorubicin microsphere in a rat tumour model. *Anti-Cancer Drugs*, **3**, 47–53.

NIIMI S, NAGAKAWA K, YOKOTA J, TSUNOKAWA Y, NISHIO K, TERASHIMA Y, SHIBUYA M, TERADA M AND SAIJO N. (1991). Resistance to drugs in NIH3T3 cells transfected with c-*myc* and c-Ha-*ras* genes. *Br. J. Cancer*, **63**, 237–241.

OGUNBIYI OA, SCHOLEFIELD JH, ROGERS K, SHARP F, SMITH JH AND POLACARZ SV. (1993). *C-myc* expression in anal squamous neoplasia. *J. Clin. Pathol.*, **46**, 23–37.

OSMAK M. (1993). Multifactorial molecular mechanisms are involved in resistance of pre-irradiated human cervix carcinoma cells to *cis*-dichlorodiammineplatinum (II) and vincristine. *Neoplasma*, **40**, 97–101.

PARIA BC, DEY SK AND ANDREWS GK. (1992). Antisense c-*myc* effects on pre-implantation mouse embryo development. *Proc. Natl Acad. Sci. USA*, **89**, 10051–10055.

RUBIN E, KHARBANDA S, GUNJI H, WEICHSELBAUM R AND KUFE D. (1992). *cis*-dichloroplatinum (II) induces c-*jun* expression in human myeloid leukemia cells: potential involvement of a protein kinase C-dependent signaling pathway. *Cancer Res.*, **52**, 878–882.

SAMBROOK J, FRITSH EF AND MANIATIS T. (1989). *Molecular Cloning: A Laboratory Manual*, 2nd edn. Cold Spring Harbor Press: Cold Spring Harbor, NY.

SCANLON KJ AND KASHANI-SABET M. (1988). Elevated expression of dTMP synthase cycle genes in cisplatin resistant A2780 cells. *Proc. Natl Acad. Sci. USA.*, **85**, 650–653.

SHAW P, BOVEY R, TARDY S, SAHLI R, SORDAT B AND COSTA J. (1992). Induction of apoptosis by wild type p53 in a human colon tumour derived cell line. *Proc. Natl Acad. Sci. USA.*, **89**, 4495–4499.

SHICHIRI M, HANSON KD AND SEDIVY JM. (1993). Effects of c-*myc* expression on proliferation quiescence and the $G_0$ to $G_1$ transition in non-transformed cells. *Cell Growth Differ.*, **4**, 93–104.

SKLAR MD AND PROCHOWNIK EV. (1991). Modulation of *cis*-platinum resistance in friend erythroleukemia cells by c-*myc*. *Cancer Res.*, **51**, 2118–2123.

SMITH DR, MYINT T AND GOH HS. (1993). Over-expression of c-*myc* in colorectal carcinoma. *Br. J. Cancer*, **68**, 407–413.

SNEDECOR GW AND COCHRAN WG. (1967). *Statistical Methods*, 6th edn. Iowa State University Press: Ames, Iowa.

SPANDIDOS DA, ZOUMPOURLIS V AND LANG JC. (1991). Cisplatin responsive sequences in the human c-*myc* promoter. *Anticancer Res.*, **11**, 1339–1342.

SPENCER CA AND GROUDINE M. (1991). Control of c-*myc* regulation. *Adv. Cancer Res.*, **56**, 1–48.

STEIN CA AND CHENG YC. (1993). Antisense oligonucleotides as therapeutic agents–is the bullet really magical? *Science*, **261**, 1004–1011.

TASHIRO H, MIYAZAKI K, OKAMURA H, IWAI A AND FUKUMOTO M. (1992). *C-myc* over-expression in primary ovarian tumours. *Int. J. Cancer*, **50**, 828–833.

TAVERNA P, HANSON J, SCANLON KJ AND HILL BT. (1994). Gene expression in X-irradiated human tumour cell lines expressing cisplatin resistance and altered DNA repair capacity. *Carcinogenesis*, **15**, 2053–2056.

TOFFOLI G, VIEL A, TUMIOTTO L, BISCONTIN G, ROSSI C AND BOIOCCHI M. (1991). Pleiotropic resistant phenotype is a multifactorial phenomenon in human colon carcinoma cell lines. *Br. J. Cancer*, **63**, 51–56.

TWENTYMAN PR, WRIGHT KA AND RHODES T. (1991). Radiation response of human lung cancer cells with inherent and acquired resistance to cisplatin. *Int. J. Radiat. Oncol. Biol. Phys.*, **20**, 217–220.

VOLM M, DRINGS P, WODRICH W AND VAN KAICK G. (1993). Expression of oncoproteins in primary human non-small cell lung cancer and incidence of metastasis. *J. Clin. Exp. Met.*, **11**, 325–329.

WATSON PH, SAFNECK JR, LE K, DUBIK D AND SHIU RP. (1993). Relationship of c-*myc* amplification to progression of breast cancer from *in situ* to invasive tumor and lymph node metastasis. *J. Natl Cancer Inst.*, **85**, 902–907.

YAGODA A AND PETRYLAK D. (1993). Cytotoxic chemotherapy for advanced hormone-resistant prostate cancer. *Cancer*, **71**, 1098–1109.

YANG Y-W AND YANG MC. (1995). Effects of c-*myc* antisense transcripts on differentiation of K562 cells. *Int. J. Oncol.*, **6**, 419–424.